# A neuroimaging measure to capture heterogeneous patterns of atrophy in Parkinson’s disease and dementia with Lewy bodies

**DOI:** 10.1016/j.nicl.2024.103596

**Published:** 2024-03-21

**Authors:** R. Bhome, S. Verdi, S.A. Martin, N. Hannaway, I. Dobreva, N.P. Oxtoby, G. Castro Leal, S. Rutherford, A.F. Marquand, R.S. Weil, J.H. Cole

**Affiliations:** a Dementia Research Centre, University College London, 8-11 Queen Square, London WC1N 3AR, United Kingdom; b UCL Centre for Medical Image Computing, Department of Computer Science, University College London, 90 High Holborn, London WC1V 6LJ, United Kingdom; c Donders Institute for Brain, Cognition, and Behavior, Radboud University, Thomas van Aquinostraat 4, 6525 GD Nijmegen, the Netherlands; d Department of Cognitive Neuroscience, Radboud University Medical Center, Kapittelweg 29, 6525 EN Nijmegen, the Netherlands; e Department of Psychiatry, University of Michigan, 4250 Plymouth Road, Ann Arbor, MI 48109, USA; f Wellcome Centre for Human Neuroimaging, University College London, 12 Queen Square, London, WC1N 3AR, United Kingdom; g Movement Disorders Consortium, National Hospital for Neurology and Neurosurgery, Queen Square, London WC1N 3BG, United Kingdom

**Keywords:** Parkinson’s disease, Dementia with Lewy bodies, Neuroanatomical normative modelling, Biomarkers, Cortical thickness, Subcortical volume

## Abstract

**Introduction:**

Parkinson’s disease (PD) and Dementia with Lewy bodies (DLB) show heterogeneous brain atrophy patterns which group-average analyses fail to capture. Neuroanatomical normative modelling overcomes this by comparing individuals to a large reference cohort. Patient-specific atrophy patterns are measured objectively and summarised to index overall neurodegeneration (the ‘total outlier count’). We aimed to quantify patterns of neurodegenerative dissimilarity in participants with PD and DLB and evaluate the potential clinical relevance of total outlier count by testing its association with key clinical measures in PD and DLB.

**Materials and methods:**

We included 108 participants with PD and 61 with DLB. PD participants were subclassified into high and low visual performers as this has previously been shown to stratify those at increased dementia risk. We generated z-scores from T1w-MRI scans for each participant relative to normative regional cortical thickness and subcortical volumes, modelled in a reference cohort (n = 58,836). Outliers (z < −1.96) were aggregated across 169 brain regions per participant. To measure dissimilarity, individuals’ Hamming distance scores were calculated. We also examined total outlier counts between high versus low visual performance in PD; and PD versus DLB; and tested associations between these and cognition.

**Results:**

There was significantly greater inter-individual dissimilarity in brain-outlier patterns in PD poor compared to high visual performers (W = 522.5; *p* < 0.01) and in DLB compared to PD (W = 5649; *p* < 0.01). PD poor visual performers had significantly greater total outlier counts compared to high (β = -4.73 (SE = 1.30); t = -3.64; *p* < 0.01) whereas a conventional group-level GLM failed to identify differences. Higher total outlier counts were associated with poorer MoCA (β = -0.55 (SE = 0.27), t = -2.04, *p* = 0.05) and composite cognitive scores (β = -2.01 (SE = 0.79); t = -2.54; *p* = 0.02) in DLB, and visuoperception (β = -0.67 (SE = 0.19); t = -3.59; *p* < 0.01), in PD.

**Conclusions:**

Neuroanatomical normative modelling shows promise as a clinically informative technique in PD and DLB, where patterns of atrophy are variable.

## Highlights

• Neuroanatomical normative modelling used for the first time in Lewy body disorders.
• Marked inter-subject heterogeneity in brain atrophy patterns in DLB and PD.
• Total outlier count is associated with clinically relevant cognitive measures in DLB.
• Total outlier count indicates deviations in brain structure in DLB and PD.

## 1 Introduction

Cognitive impairment is a core diagnostic feature of Dementia with Lewy bodies (DLB) (McKeith et al., 2017) and is common in Parkinson’s disease (PD) where almost half of patients develop dementia within ten years’ of diagnosis (Williams-Gray et al., 2013). Conventional group-level neuroimaging studies measuring brain structure in DLB and PD have yielded heterogeneous findings (Weil et al., 2019, Oppedal et al., 2019), with no consistent atrophy pattern predicting future cognitive decline (Weintraub et al., 2012, Lee et al., 2014) or correlating with symptom severity (Oppedal et al., 2019). This has limited the value of conventional neuroimaging measures as biomarkers.

A key issue in group-level analysis is between-subject heterogeneity, which results from intrinsic biological differences alongside psychosocial and environmental factors independent of the disease (Cohen-Mansfield, 2000). This has implications for case-control studies that compare group means, which only allow inferences to be made for the ‘average subject’, and treat between-subject variability as noise (Verdi et al., 2021).

To better understand the neural basis of neurodegenerative disorders such as PD and DLB, there is a need to understand between-patient heterogeneity. Neuroanatomical normative modelling is a recently established framework that maps individual patterns of variation from the expected norm (based on age and sex) for a given neuroimaging measure (Verdi et al., 2021, Marquand et al., 2016, Marquand et al., 2019). Exemplifying this approach, Rutherford and colleagues (Rutherford et al., 2022) modelled lifespan trajectories of cortical thickness and subcortical volumes using Bayesian Linear Regression based on a reference cohort of 58,836 healthy participants. Then, a new individual’s cortical and subcortical data could be plotted within each normative distribution, to quantify deviation from expected patterns. Statistical thresholds can be used to binarise the resulting z-scores to quantify neuroanatomical outliers. The number of outliers can be aggregated to provide the total outlier count, an individualised measure of overall neurodegeneration.

Neuroanatomical normative modelling has recently been applied in Alzheimer’s disease (AD) (Verdi et al., 2023, Verdi et al., 2023, Flavia et al., 2022), showing an increased number of outlier regions in AD than mild cognitive impairment (MCI) or healthy controls (Verdi et al., 2023). Importantly, total outlier count correlated with poorer cognitive performance, fluid biomarker-measures of Alzheimer’s pathology, and predicted future conversion from MCI to dementia (Verdi et al., 2023, Verdi et al., 2023). Given that neuroimaging measures in Lewy body disorders may be more neuroanatomically heterogeneous than AD (Scheltens and Korf, 2000, Mak et al., 2014), this approach may have even greater utility in Parkinson’s and DLB.

Here, we employed neuroanatomical normative modelling to investigate heterogeneity in Lewy body diseases and evaluate the potential of this technique to provide useful measures of disease severity. In PD, previous work has shown that visual performance predicts future cognitive decline, with poor visual performance associated with risk of future dementia (Zarkali et al., 2021, Hannaway et al., 2023). Here, we a) investigated differences in total outlier count between high and poor visual performers with PD; and between PD and DLB; and compared these to conventional cortical thickness analyses; b) compared patterns of dissimilarity between PD participants with high versus poor visual function; and between PD and DLB participants, and c) evaluated whether total outlier count correlated with cognitive severity in PD and DLB. We hypothesised that there would be a) significant differences in total number of regional outliers between high and poor visual performance PD groups, and in PD compared with DLB; b) greater dissimilarity in individual patients for low versus high visual performers in PD; and for DLB compared to PD. Finally, we predicted c) that greater total outlier count would be associated with poorer cognitive performance in PD and DLB.

## 2 Material and methods

### 2.1 Participants

Structural T1w-MRI data from two sites were used. The first site at University College London (UCL), included 108 participants with PD, 36 with DLB and 38 controls, from the Vision in Parkinson’s disease study (PI: Dr Weil, Queen Square Ethics Committee reference 15/LO/00476). The second site was the pseudoanonymised Alzheimer's Disease Research Center (ADRC) “8361” which contributes data to the National Alzheimer's Coordinating Center (NACC) database (Beekly et al., 2007), and included 25 participants with DLB and 127 controls. Participants from the UCL site were recruited from the National Hospital for Neurology and Neurosurgery outpatient clinics and affiliated hospitals, or from national patient support groups (Lewy Body Society and Rare Dementia Support). They were diagnosed as having PD or probable DLB if they satisfied Queen Square Brain Bank PD diagnostic criteria (Daniel and Lees, 1993) and the Dementia with Lewy Bodies Consortium Criteria (McKeith et al., 2017) respectively. Exclusions were a history of traumatic brain injury, or major co-morbid psychiatric or confounding neurological disorders; and for participants with PD, presence of dementia was also an exclusion criterion, defined using Movement Disorder Society criteria (Emre et al., 2007). All UCL participants were assessed by a neurologist (RSW) to ascertain the diagnosis of PD or DLB. Controls were recruited from spouses of patients and UCL volunteer databases. Inclusion criteria were being aged 50–80 and exclusions were the presence of past neurological or psychiatric history, or cognitive impairment on history or neuropsychological testing.

Participants from site “8361” were included if they had a structural MRI scan and met the following criteria based on descriptors available in the NACC data file (06/2022 data freeze): 1) dementia diagnosis; 2) primary or contributing cause of cognitive impairment: Lewy body disease; 3) not classed as MCI; and 4) absence of a diagnosis of PD. Controls in the NACC dataset had no evidence of cognitive impairment or history of neurological illness.

### 2.2 Clinical assessment

PD participants at the UCL site were divided into high (n = 64) and low (n = 32) visual performers based on performance on two computerised visual tasks: biological motion and the ‘Cats-and-dogs’ task (see Supplementary material). These have been described previously (Zarkali et al., 2021, Weil et al., 2017, Weil et al., 2018, Leyland et al., 2020) and shown to predict dementia and poor outcomes in Parkinson’s (Zarkali et al., 2021, Hannaway et al., 2023). We stratified the Parkinson’s group on this basis rather than on mild cognitive impairment (MCI) status because the phenomenology and presentation of PD-MCI is often heterogeneous (Muslimovic et al., 2005, Litvan et al., 2012, Yarnall et al., 2014, Weil et al., 2018) and a significant proportion of patients revert to normal cognition within five years (Pedersen et al., 2017). In contrast, patients with PD and visual dysfunction are at heightened risk of developing dementia, as shown in several longitudinal cohorts and population studies (Williams-Gray et al., 2013, Zarkali et al., 2021, Hannaway et al., 2023, Hamedani et al., 2020, Han et al., 2020).

Clinical assessment, performed on the same day as the MRI scan, included detailed neuropsychology and disease-specific measures of clinical severity. For cognition, we used the Mini-Mental State Examination (MMSE) (Folstein et al., 1975), the Montreal Cognitive Assessment (MoCA) (Nasreddine et al., 2005), which is a widely-used measure of global cognitive function in PD (Dalrymple-Alford et al., 2010) and a composite cognitive score (Zarkali et al., 2021, Hannaway et al., 2023). This combines measures across five cognitive subdomains: Stroop colour (Stroop, 1935) (attention); letter fluency (Lezak et al., 2004) (language); category fluency (Lezak et al., 2004) (executive function); word recognition (Warrington, 1984) (memory); Hooper Visual Organisation Test (Hooper, 1958) (visuo-perceptual ability), plus MoCA, averaging z-scores for each, thus providing a more comprehensive assessment of cognition. Additionally, as visuo-perceptual ability is usually affected early in DLB (McKeith et al., 2017), we specifically examined visuo-perceptual performance using the Hooper Visual Organisation Test (Hooper, 1958).

Disease-specific measures included the Movement Disorder Society Unified PD Rating Scale (MDS-UPDRS) that measures motor and non-motor domains (Goetz et al., 2008), part III of the MDS-UPDRS (MDS-UPDRS-III) to assess motor function (Goetz et al., 2008), the University of Miami PD Hallucinations Questionnaire (UM-PDHQ) to evaluate hallucinations (Papapetropoulos et al., 2008) and the Hospital Anxiety and Depression Scale (HADS) (Zigmond and Snaith, 1983) to measure depression severity.

### 2.3 MRI acquisition and processing

Structural T1w-MRI scans at UCL were acquired on a 3 T Siemens Magnetom Prisma scanner with a 64-channel head coil. Structural magnetisation prepared rapid acquisition gradient echo (MPRAGE) data were acquired using the following parameters: 1 × 1 × 1 mm voxel, TE = 3.34 ms, TR = 2530 ms, flip angle = 7°, acquisition time = 9 min. Structural T1w-MRI scans from NACC ADRC “8361” were acquired on 1.5 T GE scanners (further information on scanning parameters are available via the NACC database).

The “recon-all” function in FreeSurfer v6.0.0 (http://www.freesurfer.net) was used to process all UCL and NACC MRI data. Cortical thickness values (Destrieux parcellation; lh.aparc.a2009s.stats, rh.aparc.a2009s.stats) (Destrieux et al., 2010) and subcortical volumes (aseg.stats) were extracted. Processed images were quality controlled by visually inspecting grey and white matter boundaries, and subcortical segmentation boundaries superimposed on the corresponding structural T1-weighted image by a researcher blind to clinical status. Particular attention was paid to atrophied scans which can sometimes affect robust segmentation of brain structures.

### 2.4 Reference normative dataset

Rutherford and colleagues (Rutherford et al., 2022) modelled normative lifespan curves for cortical thicknesses across 148 regions (Destrieux parcellation) and subcortical volumes derived from Freesurfer using a warped Bayesian Linear Regression with age and sex as covariates, and accounting for site differences (Bayer et al., 2022). Bayesian linear regression with likelihood warping allows accurate modelling of non-Gaussian effects and upscaling of normative models to large cohorts (Fraza et al., 2021). Their reference cohort comprised 58,836 participants from 82 sites.

### 2.5 Applying neuroimaging normative modelling to study data

The reference normative model was recalibrated to the study datasets with an adapted transfer learning approach (Kia et al., 2022). This involved inputting control data from our two study sites into the reference normative model to generate stable parameters for cortical thicknesses and subcortical volumes, to account for residual differences in data distributions, caused by factors such as scanner differences. Z-scores were then generated for each individual with DLB or PD, per region, relative to the recalibrated reference values. All modelling steps were performed using PCNToolkit (v0.20) (Rutherford et al., 2022).

### 2.6 Statistical analysis

#### 2.6.1 Total outlier count

From the z-scores for each cortical and subcortical region generated from the normative modelling pipeline described above, outliers were defined as z-scores < -1.96. This is a commonly used threshold representing 95 % confidence that points below it differ from the mean (Fisher, 1925). This is equivalent to the *p* = 0.05 threshold for significance in frequentist statistical models, and since we are interested in atrophy, only consider lower values (i.e., the bottom 2.5 % of the population distribution) for a given neuroimaging metric. However, to ensure our findings were not driven by a particular threshold, we repeated the analysis using a more liberal outlier threshold < -1.282, to test whether this affected our findings (see Supplementary Material).

The total number of outliers across the 169 regions (148 cortical and 21 subcortical) was summed per participant to provide the total outlier count. Linear regressions, correcting for age and sex, were used to test for group differences in total outlier count between high and low visual performers with PD; and between DLB and PD. Further, subgroup analyses compared DLB participants at the UCL and NACC sites; and PD and DLB participants only at the UCL site. Group comparisons for proportion of outliers at each region were conducted using Mann-Whitney U tests and corrected for multiple comparisons using the False Discovery Rate (FDR).

#### 2.6.2 Measuring dissimilarity within and between groups

Hamming distance is widely used in information theory and reflects the dissimilarity between two strings of equal length. At each point on the strings, a distance of 1 is assigned if the symbols are different, 0 if the symbols are the same. This is summed across the length of the strings to give the Hamming distance (Hamming, 2018). Hamming distance was calculated using the vector of binarised z-scores for outliers across 169 brain regions. Participants were compared pairwise within groups, so had *n-1* Hamming distance scores ranging from 0 to 169, where *n* is their group size. Median Hamming distance scores for each participant were calculated (rather than mean, as distributions were skewed) and between-group comparisons for the median Hamming distances run, using Mann-Whitney U tests.

To visualise spatial outlier patterns of cortical thickness for each region, we calculated the proportion of participants within each group that were outliers, and mapped these onto the Destrieux atlas cortical surface using ggseg in R (Mowinckel and Vidal-Piñeiro, 2020).

#### 2.6.3 Associations between total outlier count and clinical features

Linear regressions adjusting for age and sex were used to test associations between total outlier count and composite cognitive score, MoCA and visuo-perception, measured using the Hooper Visual Organisation Test. In exploratory analyses, we tested associations with disease-specific measures including global measure of severity (MDS-UPDRS), motor severity (MDS-UPDRS-III), hallucination severity (UM-PDHQ) and depression score (HADS). Associations were tested in PD and DLB groups separately. For the DLB group we only included data from UCL where clinical severity data had been comprehensively collected.

Statistical analyses were performed in R (v4.2.2).

### 2.7 Potential outliers in total outlier count measure

One PD participant and two DLB participants (one from UCL and one from NACC) had, on data visualisation, much higher total outlier counts (45, 50 and 53, respectively) than other participants (PD range excluding outlier: 0–24; DLB: 0–38). Their brain imaging was carefully quality controlled by three authors (RB, RSW, JHC), but did not show significant structural abnormalities, acquisition, or processing errors; and clinical assessment of the UCL participants was consistent with unambiguous diagnoses (see Supplementary Table 1). We present results with and without these participants below.

### 2.8 Conventional cortical thickness analysis

We used a conventional General Linear Model (GLM) (Freesurfer v6.0) to test for regional group-level differences in cortical thickness between high and low visual performers with PD and between PD and DLB. Age and sex were used as covariates and Monte Carlo multiple comparison correction, threshold *p* < 0.05.

## 3 Results

### 3.1 Participants

We included 108 participants with PD (all from the UCL site); and 61 people with DLB (36 from UCL, 25 from the NACC site), plus 165 controls (38 from UCL, 127 from NACC), used to calibrate the reference dataset models to the study data (see Table 1 for demographic and clinical information, for further details of clinical measures in PD high and low visual performers see Supplementary Table 2).

**Table 1:** Demographics, clinical characteristics and total outlier counts.

PD vs DLB			

	PD (n = 108)	DLB (n = 61)	Statistic
Age, y	64.1 (7.8)	73.8 (6.5)	**t = -9.2; *p <* 0.01**
Male, n (%)	51 (48)	55 (90)	**χ^2^ = 27.9; *p* < 0.01**
Education, y	17.1 (2.8)	15.6 (3.4)	**W = 2483; *p* < 0.01**
Disease duration, y	4.1 (2.5)	4.3 (2.7)	W = 3393; *p* = 0.74
MMSE	29.0 (1.1)	23.3 (5.6)	W = 663.5; *p* < 0.01*
Total outlier count	3.6 (6.0)	8.7 (11.3)	β = -5.60 (SE = 1.74); *p* < 0.01*
Mean regional z-score	−0.25 (0.84)	−0.62 (0.87)	β = 0.67 (SE = 0.17); *p* < 0.01*

High vs Low visual Performers with PD			

	High (n = 62)	Low (n = 34)	Statistic
Age, y	61.5 (7.1)	68.1 (8.0)	**t = -4.02; *p* < 0.01**
Male, n (%)	31 (50)	14 (41)	χ2 = 0.38; *p* = 0.54
Education, y	16.5 (2.7)	18.2 (2.7)	**W = 720.5; *p* < 0.01**
Disease duration, y	3.7 (2.1)	4.9 (2.9)	W = 829.5, *p* = 0.54
MMSE	29.0 (1.1)	28.7 (1.2)	W = 1207; *p* = 0.22
Total outlier count	2.3 (3.5)	4.1 (5.2)	β = -4.73 (SE = 1.30); *p* < 0.01*
Mean regional z-score	−0.04 (0.42)	−0.20 (0.50)	β = 0.31 (SE = 0.10); *p* < 0.01*

DLB by site (UCL vs NACC participants)			

	UCL (n = 36)	NACC (n = 25)	Statistic
Age, y	72.9 (5.5)	75.1 (6.3)	t = -1.41; *p* = 0.17
Male, n (%)	33 (92)	22 (88)	χ2 = 0.001; *p* = 0.97
Education, y	16.0 (3.5)	15.0 (3.4)	W = 515; *p* = 0.34
Disease duration, y	3.7 (2.0)	5.3 (3.3)	**W = 315; *p* = 0.05**
MMSE	24.7 (3.4)	21.2 (7.3)	W = 547; *p* = 0.15
Total outlier count	6.3 (9.3)	12.1 (13.2)	β = -6.02 (SE = 3.00); *p* = 0.048*
Mean regional z-score	−0.39 (0.83)	−0.95 (0.81)	β = 0.61 (SE = 0.17); *p* < 0.01*

PD vs DLB at UCL site only			

	PD (n = 108)	DLB (n = 36)	Statistic
Age, y	64.1 (7.8)	72.9 (5.5)	**t = -7.54; *p* < 0.01**
Male, n (%)	51 (48)	33 (92)	**χ2 = 19.4; *p* < 0.01**
Education, y	17.1 (2.8)	16.0 (3.5)	W = 1613, *p* = 0.12
Disease duration, y	4.1 (2.5)	3.7 (2.0)	W = 2103; *p* = 0.46
MMSE	29.0 (1.1)	24.7 (3.4)	**W = 467; *p* < 0.01**
Total outlier count	3.6 (6.0)	6.3 (9.3)	β = -3.13 (SE = 1.61); *p* = 0.054*
Mean regional z-score	−0.25 (0.84)	−0.39 (0.83)	β = 0.48 (SE = 0.19); *p* < 0.01*

PD, Parkinson’s disease; DLB, Dementia with Lewy bodies; UCL, University College London; NACC, National Alzheimer’s Co-ordinating Centre.

All data are shown as mean (SD) apart from sex.

*p values were analysed by a linear regression adjusting for age and sex.

**BOLD** signifies statistically significant difference.

### 3.2 Differences in total outlier count between DLB and PD

Mean total outlier count was significantly higher in DLB (n = 61; mean = 8.7 (SD = 11.3)) compared to PD (n = 108; mean = 3.60 (SD = 6.0)) when adjusting for age and sex (PD versus DLB: β = -5.60 (SE = 1.74); t = -3.23; *p* < 0.01) and also higher in the PD low (n = 34; mean = 5.4 (SD = 8.7)) compared to high visual performers (n = 62; mean = 2.3 (SD = 3.5)), when adjusting for age and sex (β = -4.73 (SE = 1.30); t = -3.64; *p* < 0.01).

DLB participants from the NACC site (n = 25) had significantly higher numbers of outliers compared to those from the UCL site (n = 36), when adjusting for age and sex (UCL versus NACC: β = -6.02 (SE = 2.97); t = -2.02; *p* = 0.050).

Mean cortical thickness z-scores, derived from the normative modelling, showed a similar pattern of group differences as the total outlier score metric, except for DLB compared to PD group at the UCL site, where mean z-score was significantly lower in the DLB group, reflecting greater atrophy overall (Table 1).

### 3.3 Heterogeneity in patterns of outliers found between PD at-risk of dementia groups and between DLB and PD

**Fig. 1:**
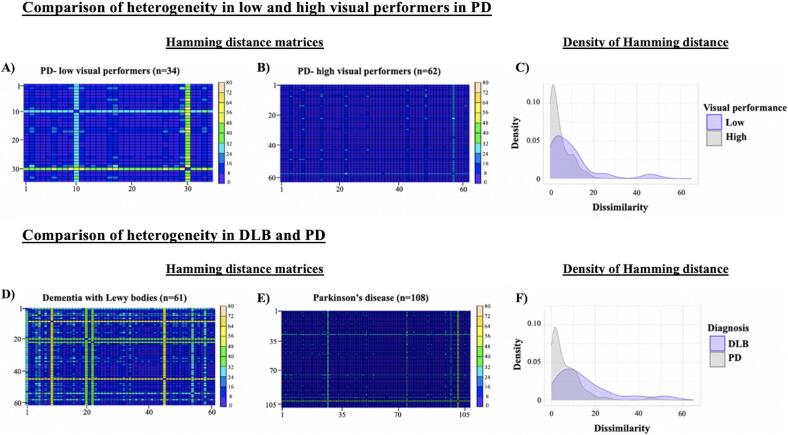
Outlier Heterogeneity. Outlier Hamming distance matrices for PD-low visual performers (A) and PD-high visual performers (B). Kernel density estimates (Y-axis) for a given Hamming distance score (X-axis) show that PD-low visual performers had more dissimilarity as evidenced by the flatter peak and longer tail compared to PD-high visual performers (C). Outlier Hamming distance matrices for the DLB (D) and PD (E) groups. Kernel density estimates (Y-axis) for a given Hamming distance score (X-axis) show that DLB participants had more dissimilarity as evidenced by the flatter peak and longer tail compared to the overall PD group (F). In A, B, D, E: dark blue / indigo represents the lower end of hamming distance scores whereby two participants are relatively similar to one another in terms of regional distribution of outliers, whereas yellow represents higher hamming distance scores, signifying greater dissimilarity. The more yellow in the plot, the greater the dissimilarity between individuals in the groups. PD, Parkinson’s disease; DLB, Dementia with Lewy bodies. (For interpretation of the references to colour in this figure legend, the reader is referred to the web version of this article.)

Dissimilarity, as measured by Hamming distance, was significantly higher in PD low visual performers (n = 34; mean = 7.1 (SD = 8.4)) compared to high visual performers (n = 62; mean = 3.3 (SD = 3.4), W = 522.5; *p* < 0.01); and higher in DLB (n = 61; mean = 12.6 (SD = 10.3)) compared to PD (n = 108; mean = 4.6 (SD = 6.0), (W = 5649; *p* < 0.01). Hamming distance matrices for between group comparisons are shown in Fig. 1.

**Fig. 2:**
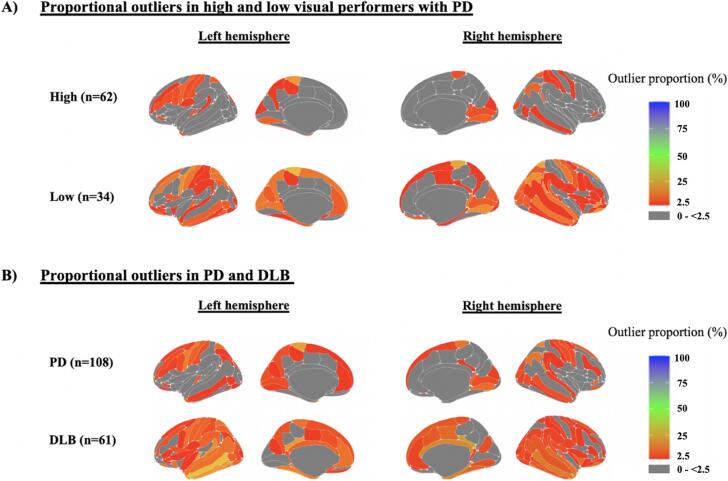
Regional maps of outliers. The proportion of participants who are outliers in a particular cortical region, mapped onto the cortical surface. A. Low visual performers (who are at-risk of Parkinson’s dementia) compared to high visual performers with PD (at lower risk of Parkinson’s dementia). Qualitatively, more regions have a higher proportion of participants with outliers in the low visual performer group. B. PD and DLB. Qualitatively, more regions have higher proportions of participants with outliers in DLB than PD. Of note, there is no one region with more than 25% of participants being outliers, highlighting the heterogeneity in cortical atrophy in DLB and PD. Grey represents regions with 0–2.5% outliers.

The proportion of regional outliers were mapped by group (Fig. 2). For low compared to high visual performers with PD, and for DLB compared to PD, there are more regions in which there are greater numbers of outliers than would be expected by chance (i.e., >2.5 %), suggesting greater heterogeneity and more widespread atrophy.

In the PD group as a whole, 125 regions out of 169 had at least one patient with an outlier. This compares with 147/169 regions in the DLB group. The region with the highest number of PD patients who were an outlier was the left paracentral lobule and sulcus region (n = 15, 13.9 %). In the DLB group the region with the highest number of outliers (15 people, only 24 % of the group) was the right posterior-dorsal part of the cingulate gyrus (dPCC). For further information on the proportion of outliers per region, and where significant regional differences between groups exist, as well as a comparison between PD low and high visual performers, see Supplementary Table 3 and Supplementary Fig. 1.

### 3.4 Total outlier counts are associated with cognitive performance in DLB and with visuospatial processing in PD

There were significant differences in several clinical measures between PD and DLB groups, with the latter more severely affected **(**Supplementary Table 4**).**

**Fig. 3:**
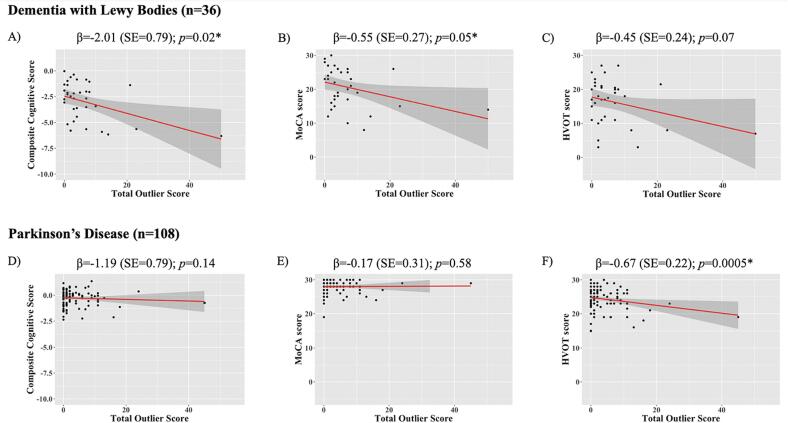
Relationship between total outlier count and cognitive measures in DLB and PD. Regression plots for the association between total outlier count (independent variable) and the following dependent variables: Composite Cognitive score, MoCA and Hooper Visual Organisation Test, in DLB (A, B, C, respectively) and PD (D, E, F, respectively). Total outlier score correlated with cognition (but not with visuo-perception); whereas in PD, total outlier score did not correlate with cognition, but did show a relationship with visuo-perception. β coefficient values, corrected for age and sex, are presented along with P values. * denotes significant association. MoCA, Montreal Cognitive Assessment; HVOT, Hooper Visual Organisation Test; DLB, Dementia with Lewy bodies; PD, Parkinson’s disease.

In DLB, there were significant associations between total outlier count and both the composite cognitive score (β = -2.01 (SE = 0.79); t = -2.54; *p* = 0.02) and MoCA (β = -0.55 (SE = 0.27), t = -2.04, *p* = 0.05), when adjusting for age and sex. There was no significant association with the Hooper Visual Organisation Test (β = -0.45 (SE = 0.24); t = -1.89; *p* = 0.068, Fig. 3**)**. There were also no significant associations with the MDS-UPDRS, MDS-UPDRS-III, UM-PDHQ or HADS (Table 2).

**Table 2:** Association of total outlier count with measures of cognitive performance and other disease specific measures.

	PD (n = 108)				DLB (n = 36)			
Attribute	beta	SE	t	*p* value^a^	beta	SE	t	*p* value^a^
Cognitive performance								
Composite Cognitive Score	−1.19	0.79	−1.51	0.14	**−2.01**	**0.79**	**−2.54**	**0.016**
MoCA	−0.17	0.31	−0.55	0.58	**−0.55**	**0.27**	**−2.04**	**0.050**
HVOT	**−0.67**	**0.19**	**−3.59**	**<0.01**	−0.45	0.24	−1.89	0.068
Disease-specific measures								
MDS-UPDRS	0.03	0.03	1.01	0.31	0.03	0.03	0.74	0.47
MDS-UPDRS Motor Score	−0.05	0.04	−1.18	0.24	0.06	0.06	0.95	0.35
UM-PDHQ	0.44	0.32	1.38	0.17	0.30	0.53	0.56	0.58
HADS depression	0.05	0.19	0.26	0.80	−0.39	0.32	−1.23	0.23

PD, Parkinson’s disease; DLB, Dementia with Lewy bodies; MoCA, Montreal Cognitive Assessment; HVOT, Hooper Visual Organisation Test; HADS, Hospital Anxiety and Depression Scale; MDS-UPDRS, Movement Disorders Society Unified Parkinson’s Disease Rating Scale; UM-PDHQ, University of Miami Hallucinations; Questionnaire; HADS, Hospital Anxiety and Depression Scale.

^a^*p* values were analysed using linear regressions adjusting for age and sex.

In **bold** results showing statistically significant associations.

In the PD group, total outlier score showed a significant association with the Hooper Visual Organisation Test (β = -0.67 (SE = 0.19); t = -3.59; *p* < 0.01), but did not show associations with global cognitive performance. Similar to DLB, no associations were found in PD between total outlier count and other disease measures (Table 2).

We repeated our analyses using a lower outlier threshold (z-score < -1.282), to ensure they were not driven by a particular threshold. The key findings were qualitatively similar to our findings using the threshold, z-score < -1.96 (Supplementary Tables 5 and 6).

Additional sensitivity analyses without the two individuals with large numbers of outlier regions showed similar results. In DLB, the association between total outlier count and poorer composite cognitive scores trended, but did not reach significance ((β = -0.94 (SE = 0.51); t = -1.83; *p* = 0.078) and MoCA (β = -0.33 (SE = 0.16); t = -2.01; *p* = 0.053). In PD, the association between total outlier count and visuo-perception (β = -0.38 (SE = 0.15); t = -2.59; *p* = 0.01) remained (Supplementary Fig. 2).

### 3.5 Group-level cortical thickness analysis is less sensitive to differences in cortical atrophy between groups

A conventional GLM approach did not find any significant clusters of differences in cortical thickness between high and low visual performers in PD. Comparing PD with DLB, there were two significant clusters in the left precentral region and one significant cluster in both the superior frontal and precentral regions on the right, signifying reduced cortical thickness in DLB compared with PD in these regions (Supplementary Table 7).

## 4 Discussion

We used neuroanatomical normative modelling to examine heterogeneity of brain atrophy in PD and DLB, to overcome the limitations of ‘group-average’ analyses. We found greater and more variable atrophy in DLB compared with PD, despite limited spatial overlap in the cortical regions affected. We showed a similar effect for people with PD at higher risk of developing dementia (low visual performance), compared to PD at low risk of dementia (high visual performers), with higher total outlier count, and greater dissimilarity in PD low visual performers than high visual performers. Importantly, conventional GLM group-average analyses did not reveal atrophy differences between these groups.

Total outlier count is agnostic to the regional location of cortical atrophy, whereas conventional GLM approaches require cortical atrophy to be in the same locations between individuals. Strikingly, total outlier count was significantly associated with severity of cognitive measures in both DLB and PD. Overall, this indicates that measures derived from neuroanatomical normative modelling may have utility in Parkinson’s and DLB.

We observed differences in total outlier count in patients at different stages in progression to dementia: in a PD dementia at-risk group (where patients did not yet have dementia); as well as in DLB. This suggests that neuroanatomical normative modelling may have clinical utility as a prognostic neuroimaging measure of disease progression in Lewy body disorders, as has been shown in Alzheimer’s disease previously (Verdi et al., 2023, Verdi et al., 2023). Importantly for its clinical application, total outlier count can be calculated based on cortical thicknesses and subcortical volume read-outs from freely-available automated pipelines for commonly acquired T1w-MRI scans.

Higher total outlier count was significantly associated with poorer global cognition (lower composite cognitive and MoCA scores) in DLB but not with a measure of visuo-perceptual processing (the Hooper test). In contrast, in PD, we did not find a relationship between composite cognitive scores and total outlier count; whereas we did find a relationship between total outlier count and visuo-perception. It is possible that the lack of relationship between cognitive measures and total outlier count in PD was due to ceiling effects in the MoCA and composite cognitive scores. In contrast, the Hooper test, which measures visual perceptual processing, may be particularly sensitive to cognitive impairment in PD because visuoperceptual and visuospatial ability are early and key cognitive domains affected in PD (Curtis et al., 2019), thus less prone to ceiling effects.

The Hamming distance enabled quantification of dissimilarity between groups, and revealed greater inter-individual heterogeneity in low compared to high visual performers with PD, and in DLB compared to PD. In both comparisons, the group associated with poorer cognitive functioning showed greater dissimilarity. This is consistent with previous work showing increased dissimilarity in Alzheimer’s compared to MCI and controls (Verdi et al., 2023). Greater dissimilarity in DLB compared to PD may relate to greater cortical involvement in DLB (Tsuboi and Dickson, 2005). Our findings highlight the benefits of considering individual differences over group-level analyses in Lewy body disease.

Normative modelling has some key advantages over alternative approaches to quantifying atrophy relative to a reference group, for example using W-score metrics, which have been previously applied to PD and DLB (Tremblay et al., 2021, Spotorno et al., 2020). The reference dataset of 58,836 used in normative modelling is around a thousand-fold larger than most W-score reference datasets, capturing much greater population variability and providing more robust estimates of deviation from control data. Further, the neuroanatomical normative modelling pipeline enables inter-individual heterogeneity to be quantified, which is not usually examined using W-score approaches.

### 4.1 Limitations

There are some limitations to consider for this work. Outliers were defined as z-scores < -1.96. This means total outliner count may fail to capture potentially relevant subthreshold levels of neurodegeneration. However, when using an alternative threshold, we found similar results, as was the case when using the mean cortical thickness z-score, which requires no threshold.

A further limitation is the differences between sites from which DLB participant data were collected. DLB data from the UCL site were collected prospectively with our study aims in mind, whereas NACC is a large relational database, where neuropsychological and clinical features information was limited. Although participants at the UCL and NACC sites did not differ in age and sex, NACC participants had longer disease duration, which may partly account for the observed increased total outlier count in that group. Alternative explanations are differences in study inclusion criteria, and testing demands on participants at the UCL site, leading to possible selection bias of less functionally impaired participants. MRI scans from the NACC site were performed on a 1.5 T scanner, and those at UCL on a 3 T scanner. However, the normative modelling pipeline is designed to help account for such differences in input data (Bayer et al., 2022) and the adapted transfer learning approach (Kia et al., 2022) allowed us to recalibrate the reference normative model based on site differences, including scanner parameters.

Finally, our DLB dataset is relatively small, although it is consistent with other imaging DLB studies (Ye et al., 2020). This may have underpowered the correlational analyses in the DLB group. DLB patients are generally frailer than those with PD and can be more challenging to assess. Recent data-sharing initiatives could enable normative modelling to be applied to larger DLB datasets from multi-site collaborations.

## 5 Conclusions

We showed that neuroanatomical normative modelling provides a new perspective on PD and DLB, which show more variable atrophy patterns between patients; and the total outlier count has potential as a clinically-useful measure of disease severity. This methodology yields personalised rather than more traditional case-control group average measures and holds promise for prognosis or treatment-response predictions for individual patients.

## 6 Disclosures

R.Bhome, S. Verdi, S.A. Martin, N. Hannaway, I. Dobreva, N.P. Oxtoby G.Castro Leal, S. Rutherford and A.F Marquand report no disclosures relevant to the manuscript.

R.S. Weil has received speaker honoraria from GE Healthcare, consulting fees from Therakind, and honoraria from Britannia.

J.H. Cole is a scientific consultant to and shareholder in BrainKey and Claritas HealthTech.

## Funding

Rohan Bhome. is supported by a Wolfson-Eisai Clinical Research Training Fellowship. Naomi Hannaway is supported by a grant by the Rosetrees and Stoneygate Trusts. Neil P Oxtoby and Gonzalo Castro Leal acknowledge support from a 10.13039/100014013UKRI Future Leaders Fellowship (MR/S03546X/1) and the 10.13039/501100000272National Institute for Health Research 10.13039/501100008721University College London Hospitals Biomedical Research Centre. A.F. Marquand gratefully acknowledges funding from the Dutch Organization for Scientific Research via a VIDI fellowship (grant number 016.156.415). Rimona S Weil is supported by a Wellcome Clinical Research Career Development Fellowship (205167/Z/16/Z).

The NACC database is funded by NIA/NIH Grant U24 AG072122. NACC data are contributed by the NIA-funded ADRCs: P30 AG062429 (PI James Brewer, MD, PhD), P30 AG066468 (PI Oscar Lopez, MD), P30 AG062421 (PI Bradley Hyman, MD, PhD), P30 AG066509 (PI Thomas Grabowski, MD), P30 AG066514 (PI Mary Sano, PhD), P30 AG066530 (PI Helena Chui, MD), P30 AG066507 (PI Marilyn Albert, PhD), P30 AG066444 (PI John Morris, MD), P30 AG066518 (PI Jeffrey Kaye, MD), P30 AG066512 (PI Thomas Wisniewski, MD), P30 AG066462 (PI Scott Small, MD), P30 AG072979 (PI David Wolk, MD), P30 AG072972 (PI Charles DeCarli, MD), P30 AG072976 (PI Andrew Saykin, PsyD), P30 AG072975 (PI David Bennett, MD), P30 AG072978 (PI Neil Kowall, MD), P30 AG072977 (PI Robert Vassar, PhD), P30 AG066519 (PI Frank LaFerla, PhD), P30 AG062677 (PI Ronald Petersen, MD, PhD), P30 AG079280 (PI Eric Reiman, MD), P30 AG062422 (PI Gil Rabinovici, MD), P30 AG066511 (PI Allan Levey, MD, PhD), P30 AG072946 (PI Linda Van Eldik, PhD), P30 AG062715 (PI Sanjay Asthana, MD, FRCP), P30 AG072973 (PI Russell Swerdlow, MD), P30 AG066506 (PI Todd Golde, MD, PhD), P30 AG066508 (PI Stephen Strittmatter, MD, PhD), P30 AG066515 (PI Victor Henderson, MD, MS), P30 AG072947 (PI Suzanne Craft, PhD), P30 AG072931 (PI Henry Paulson, MD, PhD), P30 AG066546 (PI Sudha Seshadri, MD), P20 AG068024 (PI Erik Roberson, MD, PhD), P20 AG068053 (PI Justin Miller, PhD), P20 AG068077 (PI Gary Rosenberg, MD), P20 AG068082 (PI Angela Jefferson, PhD), P30 AG072958 (PI Heather Whitson, MD), P30 AG072959 (PI James Leverenz, MD).

## Author contributions

Rohan Bhome, Rimona S Weil and James H Cole conceived the study. Rohan Bhome, Ivelina Dobreva and Naomi Hannaway collected data. Rohan Bhome, Serena Verdi, Sophie A Martin, Neil P Oxtoby and Gonzalo Castro Leal contributed to data processing and statistical analysis. Rohan Bhome wrote the first draft of the manuscript and all authors edited and agreed to the final version of the manuscript.

## CRediT authorship contribution statement

**R. Bhome:** Conceptualization, Formal analysis, Methodology, Writing – original draft, Writing – review & editing, Investigation. **S. Verdi:** Formal analysis, Methodology, Resources, Writing – review & editing. **S.A. Martin:** Data curation, Writing – review & editing. **N. Hannaway:** Data curation, Investigation, Writing – review & editing. **I. Dobreva:** Data curation, Investigation, Writing – review & editing. **N.P. Oxtoby:** Data curation, Writing – review & editing. **G. Castro Leal:** Data curation, Writing – review & editing. **S. Rutherford:** Methodology, Writing – review & editing. **A.F. Marquand:** Methodology, Writing – review & editing. **R.S. Weil:** . **J.H. Cole:** Conceptualization, Methodology, Supervision, Writing – review & editing.

## Declaration of competing interest

The authors declare that they have no known competing financial interests or personal relationships that could have appeared to influence the work reported in this paper.

## Data Availability

Data will be made available on request.

## References

[b0005] Bayer J.M.M., Dinga R., Kia S.M., Kottaram A.R., Wolfers T., Lv J. (2022). Accommodating site variation in neuroimaging data using normative and hierarchical bayesian models. Neuroimage.

[b0010] Beekly D.L., Ramos E.M., Lee W.W., Deitrich W.D., Jacka M.E., Wu J. (2007). The National Alzheimer's coordinating center (NACC) database: the uniform data set. Alzheimer Dis. Assoc. Disord..

[b0015] Cohen-Mansfield J. (2000). Heterogeneity in dementia: challenges and opportunities. Alzheimer Dis. Assoc. Disord..

[b0020] Curtis A.F., Masellis M., Camicioli R., Davidson H., Tierney M.C. (2019). Cognitive profile of non-demented Parkinson's disease: meta-analysis of domain and sex-specific deficits. Parkinsonism Relat. Disord..

[b0025] Dalrymple-Alford J.C., MacAskill M.R., Nakas C.T., Livingston L., Graham C., Crucian G.P. (2010). The MoCA well-suited screen for cognitive impairment in Parkinson disease. Neurology.

[b0030] Daniel S.E., Lees A.J. (1993). Parkinson's disease society brain Bank, London: overview and research. J. Neural Transm. Suppl..

[b0035] Destrieux C., Fischl B., Dale A., Halgren E. (2010). Automatic parcellation of human cortical gyri and sulci using standard anatomical nomenclature. Neuroimage.

[b0040] Emre M., Aarsland D., Brown R., Burn D.J., Duyckaerts C., Mizuno Y. (2007). Clinical diagnostic criteria for dementia associated with Parkinson's disease. Mov. Disord..

[b0045] Fisher R.A. (1925).

[b0050] Flavia L, Serena V, Seyed Mostafa K, Aleksandar D, Haneen H, Anna F, et al. Examining real-world Alzheimer’s disease heterogeneity using neuroanatomical normative modelling. medRxiv. 2022:2022.11.02.22281597.

[b0055] Folstein M.F., Folstein S.E., McHugh P.R. (1975). “Mini-mental state”. a practical method for grading the cognitive state of patients for the clinician. J. Psychiatr. Res..

[b0060] Fraza C.J., Dinga R., Beckmann C.F., Marquand A.F. (2021). Warped Bayesian linear regression for normative modelling of big data. Neuroimage.

[b0065] Goetz C.G., Tilley B.C., Shaftman S.R., Stebbins G.T., Fahn S., Martinez-Martin P. (2008). Movement Disorder Society-sponsored revision of the unified Parkinson's disease rating scale (MDS-UPDRS): scale presentation and clinimetric testing results. Mov. Disord..

[b0070] Hamedani A.G., Abraham D.S., Maguire M.G., Willis A.W. (2020). Visual impairment is more common in Parkinson's disease and is a risk factor for poor health outcomes. Movement Disord..

[b0075] Hamming RW. Numerical methods for scientists and engineers. Second, ed 2018.

[b0080] Han G., Han J., Han K., Youn J., Chung T.Y., Lim D.H. (2020). Visual acuity and development of Parkinson's disease: a nationwide cohort study. Mov. Disord..

[b0085] Hannaway N., Zarkali A., Leyland L.A., Bremner F., Nicholas J.M., Wagner S.K. (2023). Visual dysfunction is a better predictor than retinal thickness for dementia in Parkinson's disease. J Neurol Neurosur Ps..

[b0090] Hooper H.E. (1958).

[b0095] Kia S.M., Huijsdens H., Rutherford S., de Boer A., Dinga R., Wolfers T. (2022). Closing the life-cycle of normative modeling using federated hierarchical Bayesian regression. PLoS One.

[b0100] Lee J.E., Cho K.H., Song S.K., Kim H.J., Lee H.S., Sohn Y.H. (2014). Exploratory analysis of neuropsychological and neuroanatomical correlates of progressive mild cognitive impairment in Parkinson's disease. J. Neurol. Neurosurg. Psychiatry.

[b0105] Leyland L.A., Bremner F.D., Mahmood R., Hewitt S., Durteste M., Cartlidge M.R.E. (2020). Visual tests predict dementia risk in Parkinson disease. Neurol Clin Pract..

[b0110] Lezak M.D., Lezak M.D., HDBaLDW (2004). Neuropsychological Assessment.

[b0115] Litvan I., Goldman J.G., Troster A.I., Schmand B.A., Weintraub D., Petersen R.C. (2012). Diagnostic criteria for mild cognitive impairment in Parkinson's disease: Movement Disorder Society task force guidelines. Mov. Disord..

[b0120] Mak E., Su L., Williams G.B., O'Brien J.T. (2014). Neuroimaging characteristics of dementia with lewy bodies. Alzheimers Res. Ther..

[b0125] Marquand A.F., Rezek I., Buitelaar J., Beckmann C.F. (2016). Understanding heterogeneity in clinical cohorts using normative models: beyond case-control studies. Biol. Psychiatry.

[b0130] Marquand A.F., Kia S.M., Zabihi M., Wolfers T., Buitelaar J.K., Beckmann C.F. (2019). Conceptualizing mental disorders as deviations from normative functioning. Mol. Psychiatry.

[b0135] McKeith I.G., Boeve B.F., Dickson D.W., Halliday G., Taylor J.P., Weintraub D. (2017). Diagnosis and management of dementia with Lewy bodies fourth consensus report of the DLB consortium. Neurology.

[b0140] Mowinckel A.M., Vidal-Piñeiro D. (2020). Visualization of brain statistics with R packages ggseg and ggseg3d. Adv. Methods Pract. Psychol. Sci..

[b0145] Muslimovic D., Post B., Speelman J.D., Schmand B. (2005). Cognitive profile of patients with newly diagnosed Parkinson disease. Neurology.

[b0150] Nasreddine Z.S., Phillips N.A., Bedirian V., Charbonneau S., Whitehead V., Collin I. (2005). The Montreal cognitive assessment, MoCA: a brief screening tool for mild cognitive impairment. J. Am. Geriatr. Soc..

[b0155] Oppedal K., Ferreira D., Cavallin L., Lemstra A.W., Ten Kate M., Padovani A. (2019). A signature pattern of cortical atrophy in dementia with Lewy bodies: a study on 333 patients from the european DLB consortium. Alzheimers Dement..

[b0160] Papapetropoulos S., Katzen H., Schrag A., Singer C., Scanlon B.K., Nation D. (2008). A questionnaire-based (UM-PDHQ) study of hallucinations in Parkinson's disease. BMC Neurol..

[b0165] Pedersen K.F., Larsen J.P., Tysnes O.B., Alves G. (2017). Natural course of mild cognitive impairment in Parkinson disease: a 5-year population-based study. Neurology.

[b0170] Rutherford S., Fraza C., Dinga R., Kia S.M., Wolfers T., Zabihi M. (2022). Charting brain growth and aging at high spatial precision. Elife.

[b0175] Scheltens P., Korf E.S. (2000). Contribution of neuroimaging in the diagnosis of Alzheimer's disease and other dementias. Curr. Opin. Neurol..

[b0180] Spotorno N., Coughlin D.G., Olm C.A., Wolk D., Vaishnavi S.N., Shaw L.M. (2020). Tau pathology associates with in vivo cortical thinning in Lewy body disorders. Ann. Clin. Transl. Neurol..

[b0185] Stroop J.R. (1935). Studies of interference in serial verbal reactions. J. Exp. Psychol..

[b0190] Tremblay C., Rahayel S., Vo A., Morys F., Shafiei G., Abbasi N. (2021). Brain atrophy progression in Parkinson's disease is shaped by connectivity and local vulnerability. Brain Commun..

[b0195] Tsuboi Y., Dickson D.W. (2005). Dementia with Lewy bodies and Parkinson's disease with dementia: are they different?. Parkinsonism Relat. Disord..

[b0200] Verdi S, Rutherford S, Fraza C, Tosun D, Altmann A, Raket LL, et al. Personalising Alzheimer’s Disease progression using brain atrophy markers. medRxiv. 2023:2023.06.15.23291418.

[b0205] Verdi S., Marquand A.F., Schott J.M., Cole J.H. (2021). Beyond the average patient: how neuroimaging models can address heterogeneity in dementia. Brain.

[b0210] Verdi S., Kia S.M., Yong K.X.X., Tosun D., Schott J.M., Marquand A.F. (2023). Revealing individual neuroanatomical heterogeneity in Alzheimer disease using neuroanatomical normative modeling. Neurology.

[b0215] Warrington E.K. (1984).

[b0220] Weil R.S., Pappa K., Schade R.N., Schrag A.E., Bahrami B., Schwarzkopf D.S. (2017). The cats-and-dogs test: a tool to identify visuoperceptual deficits in Parkinson's disease. Mov. Disord..

[b0225] Weil R.S., Schwarzkopf D.S., Bahrami B., Fleming S.M., Jackson B.M., Goch T.J.C. (2018). Assessing cognitive dysfunction in Parkinson's disease: an online tool to detect visuo-perceptual deficits. Mov. Disord..

[b0230] Weil R.S., Costantini A.A., Schrag A.E. (2018). Mild cognitive impairment in Parkinson's disease-what is it?. Curr. Neurol. Neurosci. Rep..

[b0235] Weil R.S., Hsu J.K., Darby R.R., Soussand L., Fox M.D. (2019). Neuroimaging in Parkinson's disease dementia: connecting the dots. Brain Commun..

[b0240] Weintraub D., Dietz N., Duda J.E., Wolk D.A., Doshi J., Xie S.X. (2012). Alzheimer's disease pattern of brain atrophy predicts cognitive decline in Parkinson's disease. Brain.

[b0245] Williams-Gray C.H., Mason S.L., Evans J.R., Foltynie T., Brayne C., Robbins T.W. (2013). The CamPaIGN study of Parkinson's disease: 10-year outlook in an incident population-based cohort. J. Neurol. Neurosurg. Psychiatry.

[b0250] Yarnall A.J., Breen D.P., Duncan G.W., Khoo T.K., Coleman S.Y., Firbank M.J. (2014). Characterizing mild cognitive impairment in incident Parkinson disease: the ICICLE-PD study. Neurology.

[b0255] Ye R., Touroutoglou A., Brickhouse M., Katz S., Growdon J.H., Johnson K.A. (2020). Topography of cortical thinning in the lewy body diseases. Neuroimage Clin..

[b0260] Zarkali A., McColgan P., Leyland L.A., Lees A.J., Weil R.S. (2021). Visual dysfunction predicts cognitive impairment and white matter degeneration in Parkinson's disease. Mov. Disord..

[b0265] Zigmond A.S., Snaith R.P. (1983). The hospital anxiety and depression scale. Acta Psychiatr. Scand..

