# Supplementary material for: A neuroimaging measure to capture heterogeneous patterns of atrophy in Parkinson’s disease and dementia with Lewy bodies
Source: Neuroimage Clin. 2024 Mar 21;42:103596. doi: 10.1016/j.nicl.2024.103596 (PMC10995913; doi:10.1016/j.nicl.2024.103596)
Supplement: Supplementary data 1 [file mmc1.docx]

**Supplementary Material**

**Title**

**A neuroimaging measure to capture heterogeneous patterns of atrophy in Parkinson’s disease and dementia with Lewy bodies**

**Authors**

Bhome R^1,2^, Verdi S^1,2^, Martin SA^2^, Hannaway N^1^, Dobreva I^1^, Oxtoby NP^2^, Casto-Leal G, Rutherford S^5,6,7^ Marquand AF^5,6,8^, *Weil RS^1,3,4^, *Cole JH^1,2^

**Categorising PD-participants as high or low visual performers**

In brief, biological motion refers to perception of a person moving when point-lights are positioned in place of the main anatomical joints.^1^ As described previously,^2^ stimuli were generated using MATLAB and consisted of point-light figures walking (12 white dots on grey background, height: 7^o^ of visual angle, presentation time: 800ms). Position and motion were scrambled for control stimuli and motion-matched noise dots were added adaptively to increase difficulty (repetitions=225, total time=15 minutes). Using the QUEST Bayesian adaptive method,^3^ we calculated the number of noise dots tolerated to maintain 82% accuracy.

In the Cats-and-Dogs test,^4^ participants were shown greyscale images of cats and dogs skewed along the x-axis, using 11 varying degrees of skew (subtending 4° × 13° of visual angle; presentation time=280ms; 270 repetitions; total time=15 minutes). Participants were asked to discriminate whether the image was of a cat or dog (response time=3800ms). To calculate discrimination sensitivity, performance at each level of skew was calculated. Then, for each participant, a sigmoid psychophysical curve was fitted (amount of skew versus percentage correct) and skew threshold for image detection at 75% performance level was calculated.

Participants were classified as low visual performers if they performed worse than the group median performance on both tasks, capturing PD participants who consistently performed worse on higher-order visual tasks.

**Replication analysis using more liberal threshold**

Using a more liberal outlier threshold (z-score < -1.282), mean total outlier count remained significantly higher in DLB compared to PD, after adjusting for age and sex (DLB vs PD: β= -12.10 (SE=3.30); t=-3.69; *p*<0.01). It was also significantly higher in the low compared to high visual performer PD group, after adjusting for age and sex (β= -12.04 (SE=3.18); t=-3.79; *p*<0.01) **(Supplementary Table 4)**.

We also continued to show that dissimilarity, as measured by individual median Hamming distance scores, was significantly higher in DLB (n=61; mean= 40.7 (SD=13.4)) compared to PD (n=108; mean= 24.5 (SD=12.5), W=5532.5, *p*<0.01). Dissimilarity was also significantly higher in PD participants who were low visual performers (n=34; mean=29.8 (SD=15.1)) compared to high visual performers (n=62; mean=20.0 (SD=10.1), W=1586.5; *p*<0.01).

In the PD group, higher total outlier score was significantly associated with poorer composite cognitive score; and in the DLB group, it was significantly associated with worse performance on the MoCA and Hooper Visual Organisation Test **(Supplementary Table 5)**.

| **Supplementary Table 1. Clinical features of outlier participants in total outlier counts** | | |
| --- | --- | --- |
| **Total outlier count** | 45 | 50 |
| **Diagnosis** | PD | DLB |
| **Age, y** | 57 | 68 |
| **Disease duration, y** | 4 | 5 |
| **MDS-UPDRS** | 52 | 77 |
| **MDS-UPDRS motor** | 16 | 47 |
| **Composite cognitive z-score** | -0.74 | -6.31 |
| **MoCA** | 29 | 14 |
| PD, Parkinson’s disease; DLB, Dementia with Lewy bodies; MoCA, Montreal Cognitive Assessment; Hooper Visual Organisation Test, Hooper Visual Organisation Test; MDS-UPDRS, Movement Disorders Society Unified Parkinson’s Disease Rating Scale; | | |

| **Supplementary Table 2. Clinical Features of PD-high and PD-low visual performers** | | | |
| --- | --- | --- | --- |
|  | **PD-high (n=62)** | **PD-low (n=34)** | **Statistic** |
| **Age, y** | 61.5 (7.1) | 68.1 (8.0) | **t=4.03; *p*<0.01** |
| **Male, n (%)** | 31 (50) | 14 (41) | χ2=0.38; *p*=0.54 |
| **Disease Duration (years)** | 3.7 (2.1) | 4.9 (2.9) | W=829.5; *p*=0.08 |
| **Education, y** | 16.5 (2.7) | 18.2 (2.7) | **W=720.5; *p*<0.01** |
| **Composite cognitive score** | -0.07 (0.65) | -0.55 (0.88) | **W=1429; *p*<0.01** |
| **MMSE** | 29.0 (1.1) | 28.7 (1.2) | W=1207; *p*=0.22 |
| **MoCA** | 28.4 (1.6) | 27.3 (2.4) | **W=1370; *p*=0.01** |
| **HVOT** | 25.5 (2.7) | 22.8 (2.8) | **W=1638.5; *p*<0.01** |
| **Stroop colour time (sec)** | 33.9 (7.4) | 36.0 (8.3) | W=1216; *p*=0.21 |
| **Fluency letter** | 16.5 (4.8) | 17.4 (7.0) | t=0.71; *p*=0.48 |
| **Fluency category** | 22.5 (4.9) | 20.4 (6.9) | t=-1.55; *p*=0.12 |
| **Word recognition** | 24.4 (0.9) | 23.7 (1.3) | W=745.5; *p*=0.01 |
| **GNT** | 24.2 (2.5) | 23.1 (3.6) | W=904.5; *p*=0.25 |
| **MDS-UPDRS** | 42.6 (18.1) | 48.6 (24.5) | W=923.5; *p*=0.32 |
| **MDS-UPDRS motor** | 20.1 (12.7) | 20.0 (16.2) | W=971.5; *p*=0.53 |
| **UM-PDHQ** | 0.6 (1.8) | 1.1 (2.0) | W=909; *p*=0.10 |
| **HADS depression** | 3.6 (2.7) | 4.8 (3.4) | W=1252; *p*=0.13 |
| All data shown are mean (SD) except sex.  Significant differences are highlighted in **bold**.  PD, Parkinson’s disease; DLB, Dementia with Lewy bodies; GNT, Graded Naming Test; MoCA, Montreal Cognitive Assessment; Hooper Visual Organisation Test, Hooper Visual Organisation Test; HADS, Hospital Anxiety and Depression Scale; MDS-UPDRS, Movement Disorders Society Unified Parkinson’s Disease Rating Scale; UM-PDHQ, University of Miami Hallucinations; Questionnaire; HADS, Hospital Anxiety and Depression Scale | | | |

| **Supplementary Table 3. Brain regions with highest percentage of outliers in each study group** | | | |
| --- | --- | --- | --- |
| **Brain region** | **Number (%) of outliers** | **Brain region** | **Number (%) of outliers** |
| **PD- high visual performers (n=62)** | | **PD- low visual performers (n=34)** | |
| Left Paracentral lobule and sulcus | 8 (12.9) | Left Paracentral lobule and sulcus | 6 (17.7) |
| Right Superior parietal lobule | 4 (6.5) | Right Superior parietal lobule | 5 (14.7) |
| Right Lingual gyrus | 4 (6.5) | Right Paracentral lobule and sulcus | 5 (14.7) |
| Right Angular gyrus | 4 (6.5) | Right Lingual gyrus | 5 (14.7) |
| Left Precentral gyrus | 4 (6.5) | Right Angular gyrus | 5 (14.7) |
| Left Superior parietal lobule | 4 (6.5) | Left Precentral gyrus | 5 (14.7) |
| Left Transverse frontopolar gyri and sulci | 4 (6.5) | Right Orbital part of the inferior frontal gyrus | 5 (14.7) |
| Left Lingual gyrus | 4 (6.5) | Right Orbital sulci | 5 (14.7) |
|  |  |  |  |
| **PD (n=108)** | | **DLB (n=61)** | |
| Left Paracentral lobule and sulcus | 15 (13.9) | Right dPCC | 15 (24.6) |
| Right Superior parietal lobule | 11 (10.9) | Left Lateral occipito-temporal gyrus | 12 (19.7) |
| Left Precentral gyrus | 10 (9.3) | Left Middle temporal gyrus | 11 (18.0) |
| Right Paracentral lobule and sulcus | 9 (8.3) | Right Temporal pole | 11 (18.0) |
| Right Angular gyrus | 9 (8.3) | Right Lateral occipito-temporal sulcus | 11 (18.0) |
|  |  | Right Pericallosal sulcus | 11 (18.0) |
|  |  |  |  |
| Top 5 regions in terms of percentage of outliers for each study group. Where there is a tie, all regions are shown.  PD, Parkinson’s disease; DLB, Dementia with Lewy bodies; dPCC, Posterior-dorsal part of the cingulate gyrus | | | |

| **Supplementary Table 4. Clinical Features of PD and DLB groups from the UCL site** | | | |
| --- | --- | --- | --- |
|  | **PD (n=108)** | **DLB (n=36)** | **Statistic** |
| **Age, y** | 64.1 (7.8) | 72.5 (5.6) | **t=6.76; *p*<0.01** |
| **Male, n (%)** | 51 (48) | 29 (91) | **χ2=16.89; *p*<0.01** |
| **Disease duration, y** | 4.1 (2.5) | 3.7 (2) | W=2103; *p*=0.46 |
| **Education, y** | 17.1 (2.8) | 16.0 (3.5) | W=1613; *p*=0.12 |
| **Composite cognitive score** | -0.27 (0.76) | -2.93 (1.88) | **W=268; *p*<0.01** |
| **MMSE** | 29.0 (1.1) | 24.7 (3.4) | **W=467; *p*<0.01** |
| **MoCA** | 28.0 (1.9) | 20.8 (5.8) | **W=385.5; *p*<0.01** |
| **HVOT** | 24.4 (3.1) | 16.4 (6.5) | **W=476; *p*<0.01** |
| **Stroop colour time (sec)** | 35.1 (9.1) | 43.1 (13.9) | **W=2649; *p*<0.01** |
| **Fluency letter** | 16.6 (5.6) | 10.7 (5.3) | **t=-5.62; *p*<0.01** |
| **Fluency category** | 21.5 (5.7) | 9.5 (4.9) | **W=228.5; *p*<0.01** |
| **Word recognition** | 24.1 (1.2) | 21.5 (3.0) | **W=859.5; *p*<0.01** |
| **GNT** | 23.5 (3.1) | 20.0 (6.0) | **W=1166.5; *p*<0.01** |
| **MDS-UPDRS** | 45.5 (21.4) | 66.6 (27.5) | **W=2953; *p*<0.01** |
| **MDS-UPDRS motor** | 19.7 (13.6) | 34.9 (15.7) | **W=3075.5; *p*<0.01** |
| **UM-PDHQ** | 0.7 (1.8) | 4.3 (3.0) | **W=3250; *p*<0.01** |
| **HADS depression** | 4.0 (2.9) | 5.7 (3.2) | **W=2553; *p*<0.01** |
| All data shown are mean (SD) except sex.  Significant differences are highlighted in **bold**.  PD, Parkinson’s disease; DLB, Dementia with Lewy bodies; GNT, Graded Naming Test; MoCA, Montreal Cognitive Assessment; HVOT, Hooper Visual Organisation Test; HADS, Hospital Anxiety and Depression Scale; MDS-UPDRS, Movement Disorders Society Unified Parkinson’s Disease Rating Scale; UM-PDHQ, University of Miami Hallucinations; Questionnaire. | | | |

| **Supplementary Table 5. Group comparisons of total outlier counts with higher outlier threshold (z-score <-1.282)** | | | |
| --- | --- | --- | --- |
| **Overall PD vs DLB** | | | |
|  | PD (n=108) | DLB (n=61) | Statistic |
| Total outlier count | 16.1 (14.8) | 27.2 (20.4) | **β= -12.10 (SE=3.31); *p*<0.01*** |
| **High vs Low visual Performers with PD** | | | |
|  | High (n=62) | Low (n=34) | Statistic |
| Total outlier count | 12.8 (11.7) | 20.0 (18.6) | **β= -12.03 (SE=3.18); *p*<0.01*** |
| **DLB by site (UCL vs NACC participants)** | | | |
|  | UCL (n=36) | NACC (n=25) | Statistic |
| Total outlier count | 21.0 (17.7) | 36.0 (21.0)) | **β= -15.12 (SE=5.16); *p*<0.01^*^** |
| **PD vs DLB at UCL site only** | |  |  |
|  | PD (n=108) | DLB (n=36) | Statistic |
| Total outlier count | 16.1 (14.8) | 21.0 (17.7) | **β= -7.59 (SE=3.60); *p*=0.04*** |
| PD, Parkinson’s disease; DLB, Dementia with Lewy bodies; tOC, total outlier count; UCL, University College London; NACC, National Alzheimer’s Co-ordinating Centre  All data are shown as mean (SD).  ^*^*p* values were analysed by a linear regression adjusting for age and sex  **BOLD** signifies statistically significant difference | | | |

| **Supplementary Table 6. Association of total outlier count with measures of cognitive performance and other disease specific measures (replication analysis with threshold z-score < -1.282)** | | | | | | | | |
| --- | --- | --- | --- | --- | --- | --- | --- | --- |
|  | PD (n=108) | | | | DLB (n=36) | | | |
| Attribute | beta | SE | t | *p* value^a^ | beta | SE | t | *p* value^a^ |
| Cognitive performance | | | | | | | | |
| Composite Cognitive Score | -1.70 | 1.96 | -0.37 | 0.39 | **-3.46** | **1.55** | **-2.23** | **0.03** |
| MoCA | -0.17 | 0.31 | -0.55 | 0.58 | **-1.13** | **0.51** | **-2.21** | **0.03** |
| HVOT | **-1.28** | **0.48** | **-2.68** | **<0.01** | -0.55 | 0.47 | -1.17 | 0.25 |
| Disease-specific measures | | | | | | | | |
| MDS-UPDRS | 0.37 | 0.66 | 0.57 | 0.57 | 0.12 | 0.11 | 1.05 | 0.30 |
| MDS-UPDRS Motor Score | -0.13 | 0.10 | -1.26 | 0.21 | 0.28 | 0.19 | 1.43 | 0.16 |
| UM-PDHQ | 0.83 | 0.79 | 1.05 | 0.30 | 0.29 | 1.02 | 0.28 | 0.78 |
| HADS depression | 0.21 | 0.48 | 0.45 | 0.65 | -0.68 | 1.04 | -0.65 | 0.52 |
| PD, Parkinson’s disease; DLB, Dementia with Lewy bodies; MoCA, Montreal Cognitive Assessment; HVOT, Hooper Visual Organisation Test; HADS, Hospital Anxiety and Depression Scale; MDS-UPDRS, Movement Disorders Society Unified Parkinson’s Disease Rating Scale; UM-PDHQ, University of Miami Hallucinations; Questionnaire; HADS, Hospital Anxiety and Depression Scale.  ^a^*p* values were analysed using linear regressions adjusting for age and sex.  In **bold** results showing statistically significant associations | | | | | | | | |

| **Supplementary Table 7. Significant clusters for the left and right hemisphere comparing PD and DLB cortical thicknesses.** | | | | | | |
| --- | --- | --- | --- | --- | --- | --- |
|  | **Region** | **Cluster size (mm^2^)** | **MNI Coordinates** | | | **CWP** |
|  |  |  | **x** | **y** | **z** |  |
| **Left hemisphere** |  |  |  |  |  |  |
|  | Precentral | 103.33 | -29.5 | -13.1 | 58.9 | 0.013 |
|  | Precentral | 90.01 | -33.7 | -16.1 | 41.1 | 0.023 |
| **Right hemisphere** |  |  |  |  |  |  |
|  | Superiorfrontal | 110.67 | 16.4 | -3.5 | 66.7 | 0.011 |
|  | Precentral | 89.84 | 30.7 | -12.4 | 52.0 | 0.020 |
| *p* values from the Monte carlo simulation and clustering as cluster wise probability (CWP), resulting from the vertex-wise comparison of cortical thickness between PD and DLB participants.  MNI, Montreal Neurological Institute; PD, Parkinson’s disease; DLB, Dementia with Lewy bodies | | | | | | |


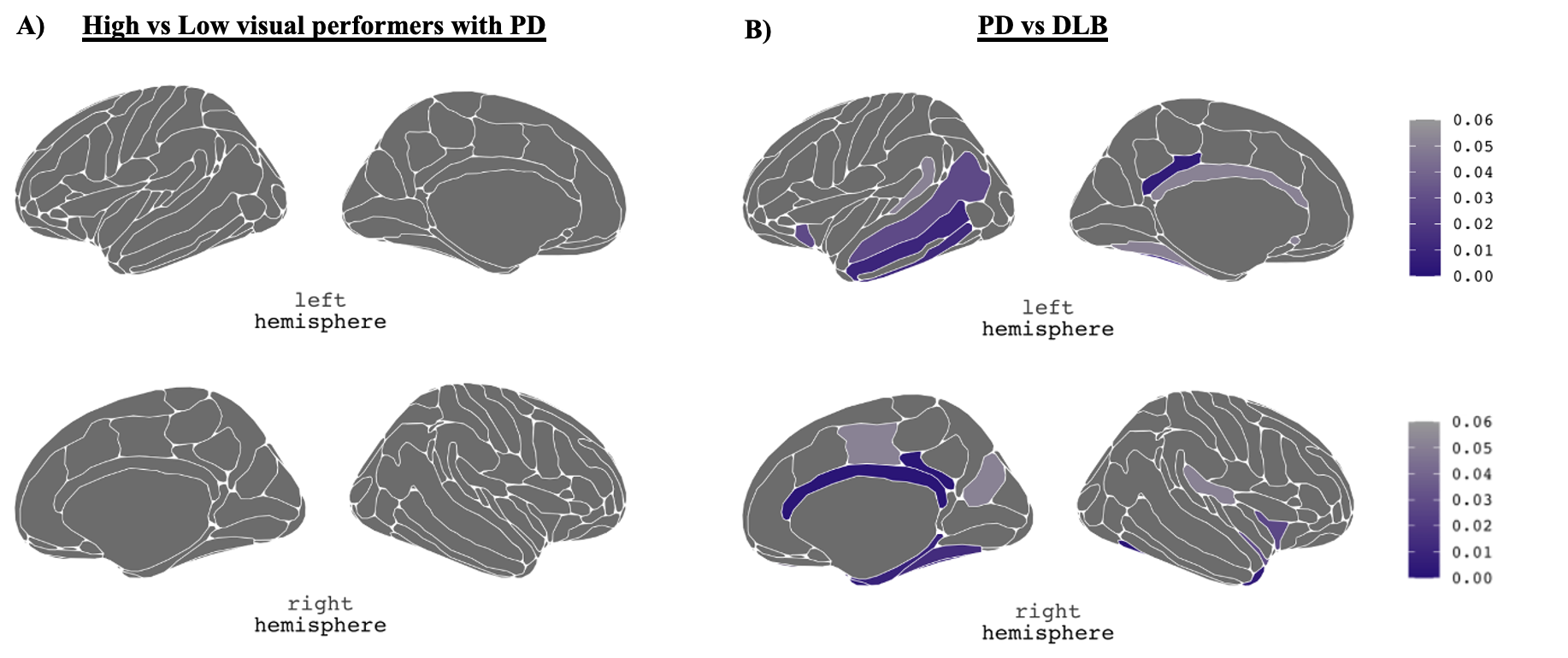


**Supplementary Figure 1. Regional Outliers.** Comparing high and low visual performers with PD, there were no FDR corrected significant differences in the proportion of participants who were outliers in any cortical region (A); however there were sixteen regions where the proportion of participants who were outliers was significantly greater for DLB than PD (FDR *p* value <0.05): right dPCC, right lateral occipito-temporal sulcus, right pericallosal sulcus, left dPCC, right temporal pole, left lateral occipito-temporal gyrus, right parahippocampal gyrus, right lateral occipito-temporal gyrus, left middle temporal gyrus, left inferior temporal gyrus, right medial occipito-temporal sulcus and lingual sulcus, right short insular gyri, right hippocampus, left superior temporal sulcus,left anterior segment of the circular sulcus of the insula, right planum polare of the superior temporal gyrus

PD, Parkinson’s disease; DLB, Dementia with Lewy bodies; dPCC, Posterior-dorsal part of the cingulate gyrus.


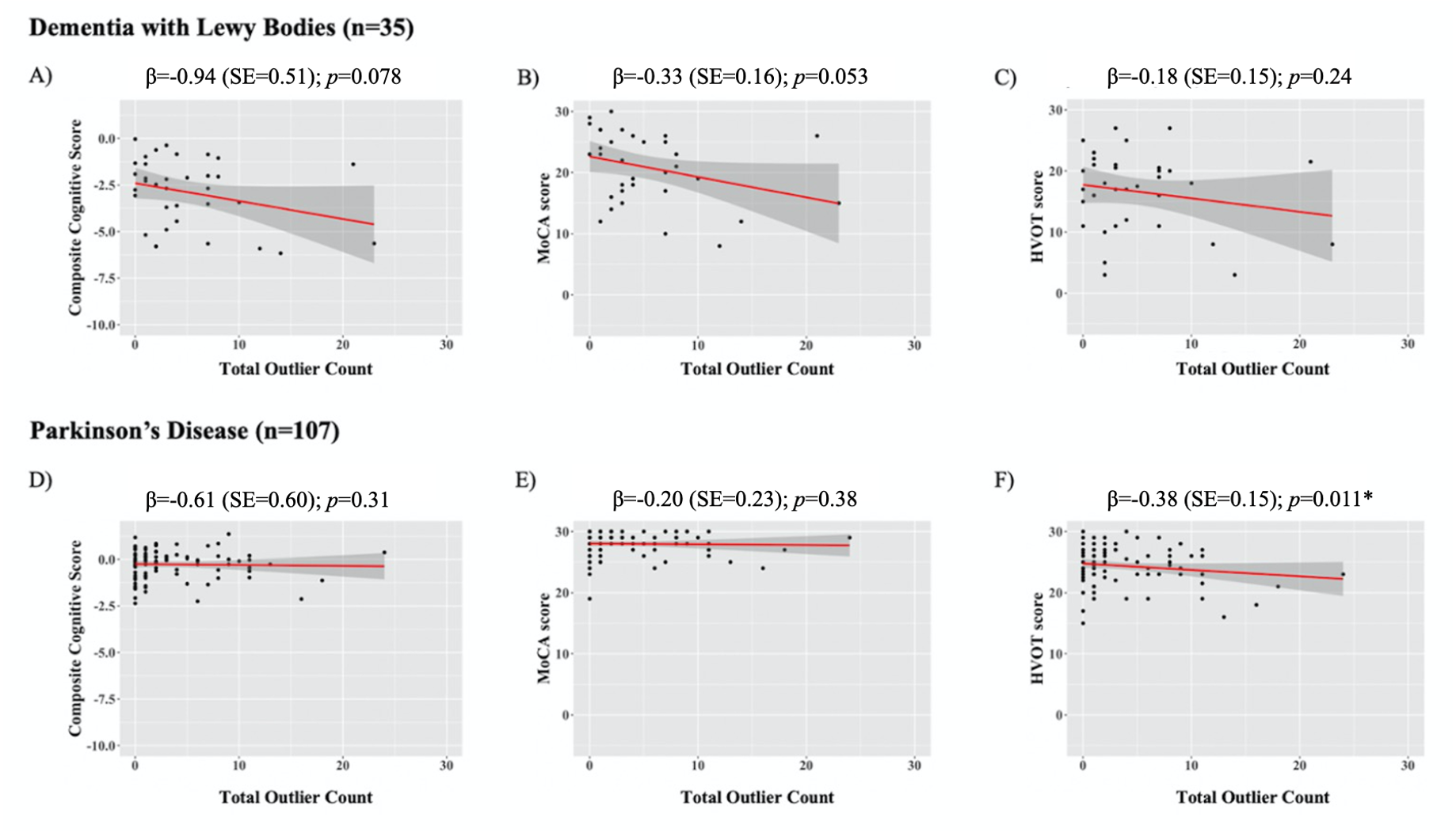


**Supplementary Figure 2. Relationship between total outlier count, with potential outlying scores excluded, and cognitive measures in DLB and PD.** Regression plots for the association between total outlier count (independent variable) and the following dependent variables: Composite Cognitive score, MoCA and HVOT, in DLB (A, B, C , respectively) and PD (D, E, F, respectively).

β coefficient values, corrected for age and sex, are presented along with P values. * denotes significant association.

MoCA, Montreal Cognitive Assessment; HVOT, Hooper Visual Organisation Test; DLB, Dementia with Lewy bodies; ; PD,. Parkinson’s disease

**Supplementary References**

1. Saygin AP. Superior temporal and premotor brain areas necessary for biological motion perception. Brain. 2007;130(Pt 9):2452-61.

2. Leyland LA, Bremner FD, Mahmood R, Hewitt S, Durteste M, Cartlidge MRE, et al. Visual tests predict dementia risk in Parkinson disease. Neurol Clin Pract. 2020;10(1):29-39.

3. Watson AB, Pelli DG. QUEST: a Bayesian adaptive psychometric method. Percept Psychophys. 1983;33(2):113-20.

4. Weil RS, Pappa K, Schade RN, Schrag AE, Bahrami B, Schwarzkopf DS, et al. The Cats-and-Dogs test: A tool to identify visuoperceptual deficits in Parkinson's disease. Mov Disord. 2017;32(12):1789-90.
